# CVIGAN: Contrastive Visible-to-Infrared Generative Adversarial Network with ConvNeXtV2 Multi-Scale Frequency-Aware Attention

**DOI:** 10.3390/s26196223

**Published:** 2026-09-30

**Authors:** Chengyi Wang, Jin Lin, Ting Liu, Xiangyi Lu, Ziheng Wang, Kai Zheng

**Affiliations:** 1 College of Marine Electrical Engineering, Dalian Maritime University, Dalian 116026, China; 15804213244@163.com (C.W.); luxiangyi@dlmu.edu.cn (X.L.); 18241122752@139.com (Z.W.); kzh@dlmu.edu.cn (K.Z.); 2 Dalian Science Test and Control Technology Institute, Dalian 116013, China; linjinmh_20008@126.com

**Keywords:** visible-to-infrared image generation, ConvNeXtV2, multi-scale frequency-aware attention, contrastive generative adversarial network

## Abstract

**Highlights:**

**What are the main findings?**

- CVIGAN is proposed in this paper, which is an unsupervised generative adversarial network for unpaired visible-to-infrared generation. A ConvNeXtV2 generator embedded with the multi-scale frequency-aware feature enhancement block (MFEB) is built to suppress illumination-color interference and remedy blurry contours and incomplete small-target artifacts.
- A multi-scale discriminator and a weighted multi-component composite loss function are tailored. They jointly impose local detail constraints and global infrared grayscale distribution regularization, alleviating visual pattern distortion and semantic misalignment in generated infrared outputs.

**What are the implications of the main findings?**

- The proposed CVIGAN produces realistic infrared images, which can provide reliable feature support for downstream vision tasks including object detection and segmentation.

**Abstract:**

Existing unsupervised visible-infrared image generation methods struggle to produce reasonable target thermal appearances. Consequently, it is challenging to generate infrared images with consistent structural details. To address this limitation, this paper proposes CVIGAN: Contrastive Visible-to-Infrared Generative Adversarial Network with ConvNeXtV2 Multi-Scale Frequency-Aware Attention. Specifically, ConvNeXtV2 is leveraged to construct the encoder of the generator, and a multi-scale frequency-aware attention feature enhancement block (MFEB) is integrated to enhance the feature representation capability. Secondly, multi-scale discriminators are elaborately designed to simultaneously capture local fine-grained details and global structural information of target-domain images. Finally, a composite loss function is formulated by combining multi-layer contrastive loss, unidirectional cycle-consistency loss, identity loss, and least-squares adversarial loss, which enforces cross-domain feature alignment and restricts the quality of generated outputs. The effectiveness of the proposed method is validated through experiments on the unpaired AVIID3, M3FD, and a self-built dataset. The generated infrared images achieve notably enhanced image quality. In addition, the robustness of CVIGAN is validated through cross-scene and cross-resolution generalization experiments, where favorable performance is maintained under unseen scenes and high-resolution input conditions. Thus, the generated infrared images are validated to effectively enhance downstream infrared object detection tasks, offering a more efficient solution for infrared image acquisition and analysis in real-world applications.

## 1. Introduction

Benefiting from the unique advantages of strong anti-interference capability, effective smoke penetration, and stable performance under low-light conditions, infrared imaging technology has been widely employed in a variety of critical fields, ranging from industrial monitoring [1,2] and autonomous driving [3,4] to military reconnaissance [5] and disaster assessment [6]. Infrared images can directly reflect the thermal radiation intensity distribution of targets. Their grayscale values are strongly correlated with physical properties such as material composition and surface temperature. Thus, it can reveal target features that are indiscernible to visible-light cameras. However, the acquisition of high-quality infrared images relies on specialized thermal imagers, which suffer from high costs during data collection procedures. Accordingly, large-scale public infrared datasets are still lacking, especially for maritime scenarios, where relevant data resources are extremely scarce. Consequently, the research and validation of data-driven infrared image processing algorithms are severely constrained. Therefore, infrared image generation technology is considered a viable solution to the problems described above.

Traditional infrared image generation is achieved by a mechanism modeling approach based on the physical theory of radiative heat transfer. Infrared grayscale values are computed pixel by pixel, using physical parameters such as target material properties and temperature, through which high physical reliability is ensured. However, accurate scene geometry and material parameters are required by this method, which renders the modeling process extremely costly. With the rapid advancement of deep network-based image style transfer techniques in recent years, great research interest has been attracted to visible-to-infrared image generation at the intersection of computer vision and infrared technology. Visible-light images are widely available, high-resolution, and easily accessible, while high-quality infrared images can be generated from visible data by means of deep learning algorithms. This is expected to overcome the above data bottleneck [7].

Two main categories of approaches have been researched for visible-to-infrared image generation. The first category is constituted by supervised deep convolutional transformation networks, such as image translation frameworks Pix2Pix [8] and Pix2PixHD [9]. Strictly paired visible-infrared annotated samples are required by these methods. The collection of large-scale paired data is difficult for complex scenarios, including maritime aerial photography and urban roads, which limits practical engineering deployment. The second category is unsupervised Generative Adversarial Networks (GANs) [10]. Only unpaired images from the visible and infrared domains are required, and this approach has been recognized as the mainstream solution for cross-modal image generation.

In recent years, extensive studies have been conducted by numerous researchers on the unpaired mapping task from the visible domain to the infrared domain. A Siamese semantic loss mechanism was introduced by Wang et al. [11] into the CycleGAN framework to generate thermal infrared textures for traffic scenarios. However, the reliability of the method is considered to be insufficient due to the absence of paired real images for quantitative evaluation. CUT (Contrastive Learning for Unpaired Image-to-Image Translation) [12] was proposed and incorporates contrastive learning to optimize the image translation pipeline. Cross-domain mapping is learned by maximizing feature similarity between source and target images, such that the features of generated target-domain images are approximated to those of real target-domain samples. UNSB [13] was proposed by integrating regularization techniques with contrastive learning, and a multi-iteration strategy was adopted to further preserve fine details during cross-domain image translation. Aiming at the performance degradation of unpaired visible-infrared generation methods in the mid-wave infrared band, CUT P2PGM [14] was proposed, in which a task-aware unpaired translation framework was conducted, combining pseudo-pair generation and semantic consistency constraints. Besides, robust domain adaptability has been demonstrated for practical applications. DGCGAN [15] was presented with a dual geometric cycle generative adversarial network. Geometric consistency constraints and cycle-consistency constraints are unified, and disentangled structure-style representation learning is utilized to support one-to-many visible-to-infrared image generation.

However, multiple inherent drawbacks have been identified in existing unsupervised GAN methods for visible-infrared generation, which are intrinsically associated with network module designation and loss function construction:

1. Insufficient feature extraction capability of generators: the encoders are mostly constructed with residual blocks in typical frameworks represented by CycleGAN and CUT, which yield limited receptive fields. Irrelevant interferences introduced by color and illumination in visible images are difficult to be effectively filtered out by such structures and multi-scale frequency-aware feature fusion mechanisms are also lacked. The loss of shallow semantic information during decoding consequently leads to generated infrared images with blurry outlines, incomplete small targets, and distorted texture details.
2. Lack of global constraints in discriminators: Only local pixel-level discrepancies are focused on by traditional single-scale discriminators, while the global grayscale distribution and overall layout of real infrared images are ignored. As a result, the generated infrared images suffer from unreasonable overall grayscale layout and inconsistent visual style compared with real infrared samples.
3. Simplified and unbalanced loss functions: Adversarial loss and cycle-consistency loss are adopted by existing methods without identity feature correction terms. The translated images are prone to brightness and texture shifts, and the loss design cannot simultaneously constrain global structural integrity and local details.
  The aforementioned problems directly cause visual feature distortion and semantic misalignment in generated infrared images, making it difficult to support downstream tasks such as object detection [16], object tracking [17,18], and image segmentation [19,20]. Therefore, improvements from three perspectives are urgently required, namely the generator, discriminator, and composite loss function.

Based on the above analysis, this paper proposes an unsupervised generative adversarial network named CVIGAN (Contrastive Visible-to-Infrared Generative Adversarial Network) for visible-to-infrared image generation. The main contributions of this paper are summarized as follows:

1. A ConvNeXtV2-based generator is constructed. ConvNeXtV2 is adopted as the downsampling backbone, and a multi-scale frequency-aware feature enhancement block (MFEB) is embedded. Leveraging the large convolution kernels of ConvNeXtV2, together with multi-branch high-low frequency-aware attention inside MFEB, our generator aggregates target thermal radiation features to address incomplete small objects and blurry textures.
2. A multi-scale discriminator is designed to enhance the fine details and photorealism of generated images. By efficiently capturing both global and local image information, the discriminator guarantees that the generated infrared images match real infrared samples in terms of visual quality and infrared style distribution.
3. A weighted multi-component composite loss function is constructed. Multi-layer PatchNCE contrastive loss is introduced to achieve cross-domain semantic alignment of targets, which collaborates with least-squares adversarial loss, unidirectional cycle loss, and identity loss to jointly constrain the network and mitigate spatial misalignment of targets, visually unreasonable thermal patterns, and brightness shifts.

To address the above-mentioned drawbacks of existing unsupervised visible-to-infrared generation methods, this paper proposes CVIGAN, a contrastive generative adversarial network equipped with ConvNeXtV2 and multi-scale frequency-aware attention. The detailed network architecture and composite loss function are elaborated in Section 2. Extensive comparative experiments, ablation studies, and generalization-ability evaluations are presented in Section 3, followed by conclusions in Section 4.

## 2. Proposed Method

**Figure 1:**
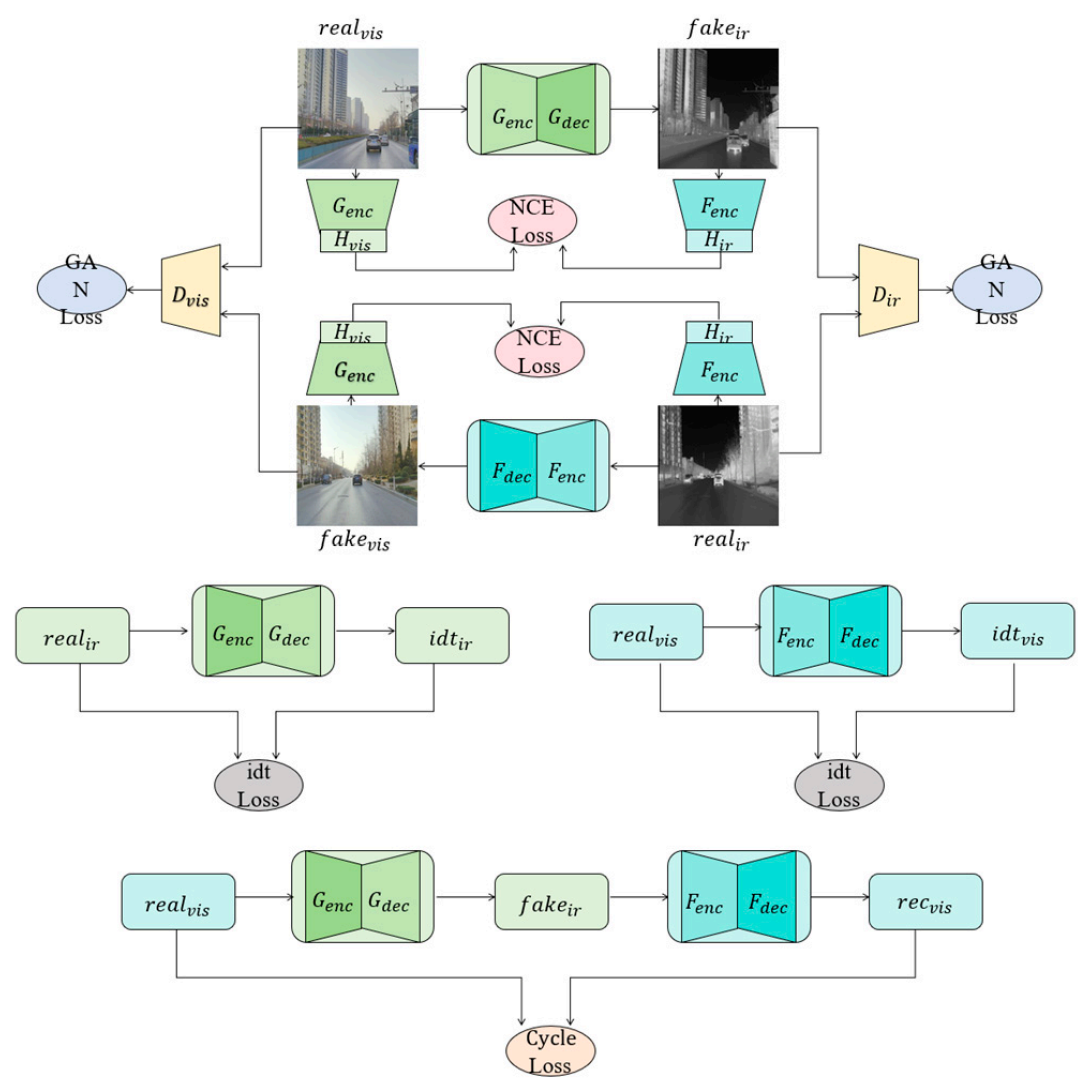
Overall Network Architecture of CVIGAN. Dual-branch generators complete bidirectional visible-infrared translation under multi-loss constraints including adversarial, contrastive, identity, and cycle-consistency loss.

Existing unsupervised image generation methods mainly focus on the transformation of visual styles such as color and texture. However, it remains challenging to reconstruct plausible infrared thermal appearances while preserving precise scene structures. Therefore, a contrastive learning mechanism is introduced in this paper, and a generative adversarial network named CVIGAN (Contrastive Visible-to-Infrared Generative Adversarial Network) is proposed for visible-to-infrared image generation. The overall architecture of the proposed CVIGAN is shown in Figure 1. CVIGAN consists of two pairs of generators and discriminators, namely {G, D_ir_} and {F, D_vis_}. G performs visible-to-infrared generation, and Dir constrains generated outputs to fit the infrared domain distribution; F realizes infrared-to-visible generation, while D_vis_ guarantees the generated images satisfy the visible domain distribution. Both generators G and F are composed of encoders G_enc_, F_enc_ and decoders G_dec_, F_dec_.

The formulation for generating infrared images is presented in Equation (1), where *real_vis_* denotes the input real visible-light image, *fake_ir_* represents the synthesized infrared image, and the generator G is constructed by serially connecting the encoder Genc and the decoder *G_dec_*.

*fake_ir_* = *G*(*real_vis_*) = *G_dec_*(*G_enc_*(*real_vis_*)) (1)

During the cross-domain image generation process, multi-layer feature representations are extracted from the encoder of input images. An MLP network is adopted to project the extracted features into a shared embedding space, based on which the multi-layer contrastive loss between input and output images is calculated. Furthermore, an identity loss function and a unidirectional cycle-consistency loss function are introduced to regularize the generation of infrared images.

### 2.1. Generator Architecture

**Figure 2:**
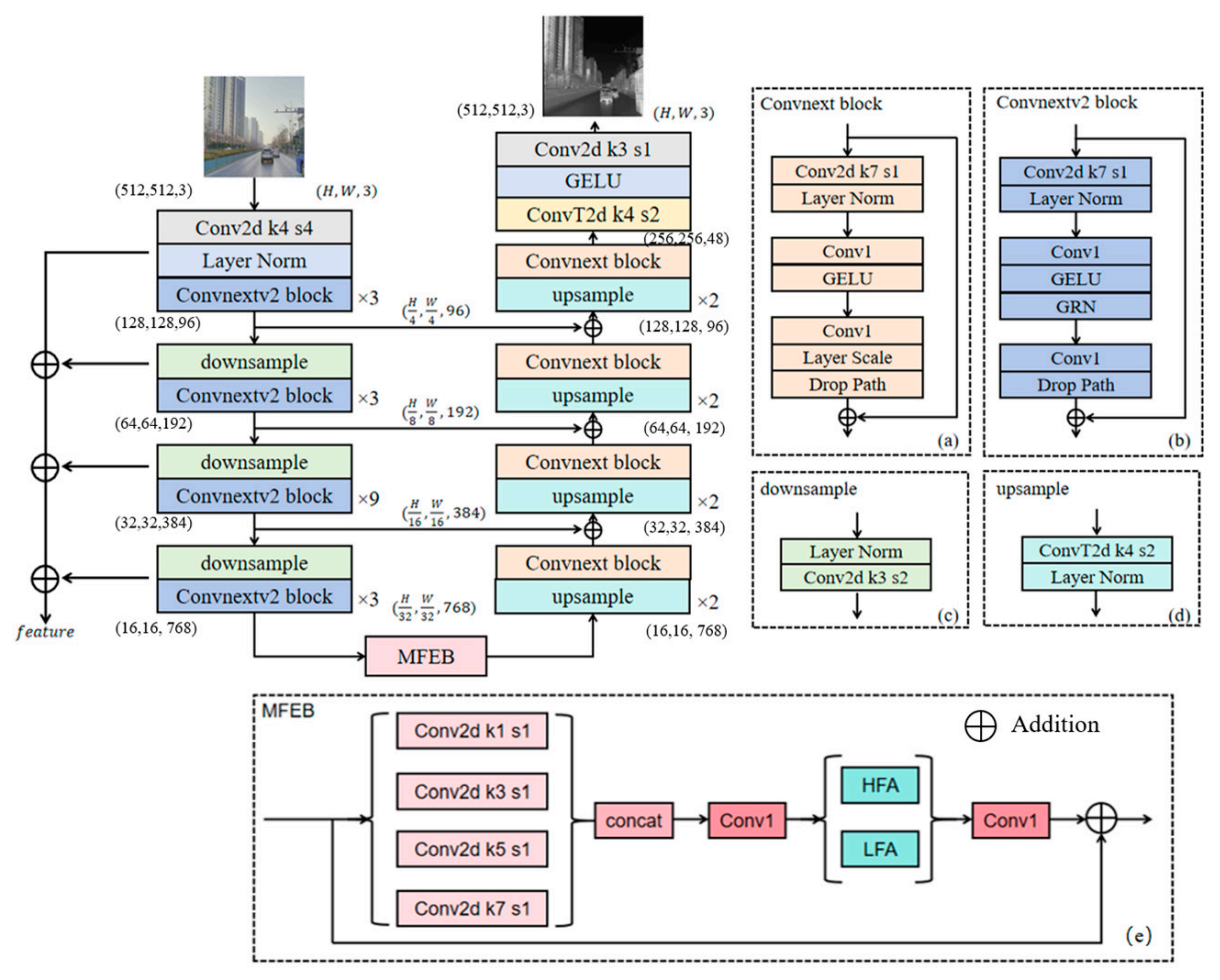
Diagram of multi-scale frequency-aware attention-enhanced ConvNeXt generator. The encoder adopts stacked ConvNeXtV2 blocks for multi-scale feature extraction, and the proposed MFEB module integrates multi-scale convolution and dual-branch high/low frequency-aware attention to strengthen feature representation before decoder reconstruction. (**a**) ConvNeXt block; (**b**) ConvNeXtV2 block; (**c**) Downsampling module; (**d**) Upsampling module; (**e**) Multi-scale Frequency-aware Attention Feature Enhancement Block (MFEB).

ConvNeXtV2 [21], a state-of-the-art image classification network, is adopted as the encoder backbone of the generator. As depicted in Figure 2, the input visible images(*real_vis_*) are downsampled by the encoder branch of the generator via convolutions with strides of 4 and 2. ConvNeXt_blockV2 modules are utilized to extract content features, and semantic features from every encoder layer are reserved for calculating multi-layer contrastive loss. In the decoder branch, transposed convolutions with strides of 4 and 2 are deployed for up-sampling to reconstruct high-resolution feature maps. Meanwhile, skip connections [22] are embedded to fully exploit semantic information and deep feature representations extracted from the encoder. Finally, transposed convolutions and channel-wise convolutions are adopted to progressively up-sample and adjust channel dimensions, yielding the final infrared output image.

#### 2.1.1. Encoder Architecture

The ConvNeXtV2 network has been employed to extract features from input images, as illustrated in Figure 2a. To expand the receptive field, large 7 × 7 convolution kernels are adopted by each ConvNeXt_blockV2.

Specifically, the Global Response Normalization (GRN) layer is an internal component of the ConvNeXtV2 block. GRN operates in three sequential steps. Firstly, global feature aggregation is performed by compressing the feature map of each channel into a single vector via the L2 norm; secondly, feature normalization transforms the aggregated vector using a standard divisive normalization function; third, feature recalibration is accomplished by modulating the original feature maps with the normalized vectors. Note that GRN introduces no additional trainable parameters and supports training with arbitrary batch sizes. To balance model parameter overhead, we follow the experimental configuration of ConvNeXtV2-Thin. The channel dimensions of four stages are set to 96, 192, 384 and 768, and the stacking ratio of ConvNeXt blocks within each stage is 3:3:9:3. In addition, a dedicated down-sampling module is designed, as shown in Figure 2c. It consists of a normalization layer followed by a 3 × 3 convolutional layer with stride = 2 and padding = 1.

#### 2.1.2. Multi-Scale Feature Enhancement Module

To further aggregate global feature information, a Multi-Scale Feature Enhancement Block (MFEB) with a frequency-aware attention mechanism is inserted between the encoder and decoder. As illustrated in Figure 2e, a parallel multi-branch depthwise separable convolution architecture with 1 × 1, 3 × 3, 5 × 5, and 7 × 7 convolution kernels is first adopted to construct a feature pyramid with diverse receptive fields. Each convolution branch uses stride = 1 and padding = k//2(where k denotes kernel size). Every branch outputs feature tensors that retain identical spatial height H and width W as the input feature map, with tensor shape [B, dim, H, W] for each branch output. Subsequently, the four groups of feature maps are concatenated along the channel dimension. The concatenated tensor yields a shape of [B, 4 × dim, H, W]. Afterward, a 1 × 1 convolution is deployed to perform channel dimension reduction and information fusion on the concatenated high-dimensional features, compressing the multi-scale features into feature maps with a unified dimension of [B, dim, H, W]. Multi-scale pooling is performed on the input feature maps to generate four feature maps with different resolutions for feature reconstruction. Subsequently, the four groups of feature maps are concatenated along the channel dimension to achieve multi-scale feature aggregation. Afterward, a 1 × 1 convolution is deployed to perform channel dimension reduction and information fusion on the concatenated high-dimensional features, compressing the multi-scale features into feature maps with a unified dimension. Next, a dual-path attention mechanism consisting of low-frequency-aware attention and high frequency-aware attention is embedded into the module. To well balance the model representation capability and computational cost, convolutions are utilized to extract local fine-grained details of high frequency-aware features, while global average pooling is adopted to capture global contextual information from low frequency-aware features. With this parallel dual-path attention, the importance of local fine textures and global structural information can be adaptively weighted. The attention-modulated multi-scale features are fused via channel-wise concatenation, followed by a 1 × 1 convolution for feature reorganization. Finally, a residual connection is employed to fuse the enhanced features with the original input of the module, ensuring that the output feature map shares identical channel number, width, and height with the input feature map.

#### 2.1.3. Decoder Architecture

In the decoder branch, skip connections are adopted to fully exploit semantic information and deep feature representations extracted from the encoder. Two stacked ConvNeXt blocks are deployed at each stage for image reconstruction. As presented in Figure 2b, the extracted features need to be sufficiently reused during image reconstruction. Thus, Layer Normalization (LN) layers are incorporated into each ConvNeXt block to constrain activations within a stable range. This design avoids vanishing and exploding gradients caused by excessively large or small activations, and suppresses artifacts and noise that may emerge during image generation. Consistent with the encoder design, a dedicated up-sampling module is elaborated, as shown in Figure 2d. It consists of a normalization layer and a transposed convolution layer with a 4 × 4 kernel, stride of 2, and padding of 2.

### 2.2. Design of Multi-Scale Discriminator

Conventionally, input images are processed by stacked convolutional layers within a discriminator and eventually compressed into one-dimensional feature vectors to quantify the similarity between real images(*real_ir_*) and synthetic images(*fake_ir_*). Nevertheless, conventional discriminative schemes are dependent on fixed-size sliding windows. Excessive importance is placed on local feature discrepancies while global feature distributions are neglected. To enable the discriminator to simultaneously capture local fine-grained features and global structural information and produce more reliable discriminative outputs that guide the generator to synthesize high-quality synthetic images(*fake_ir_*) with consistent global layouts and delicate details, a multi-scale discriminator is adopted in this work.

**Figure 3:**
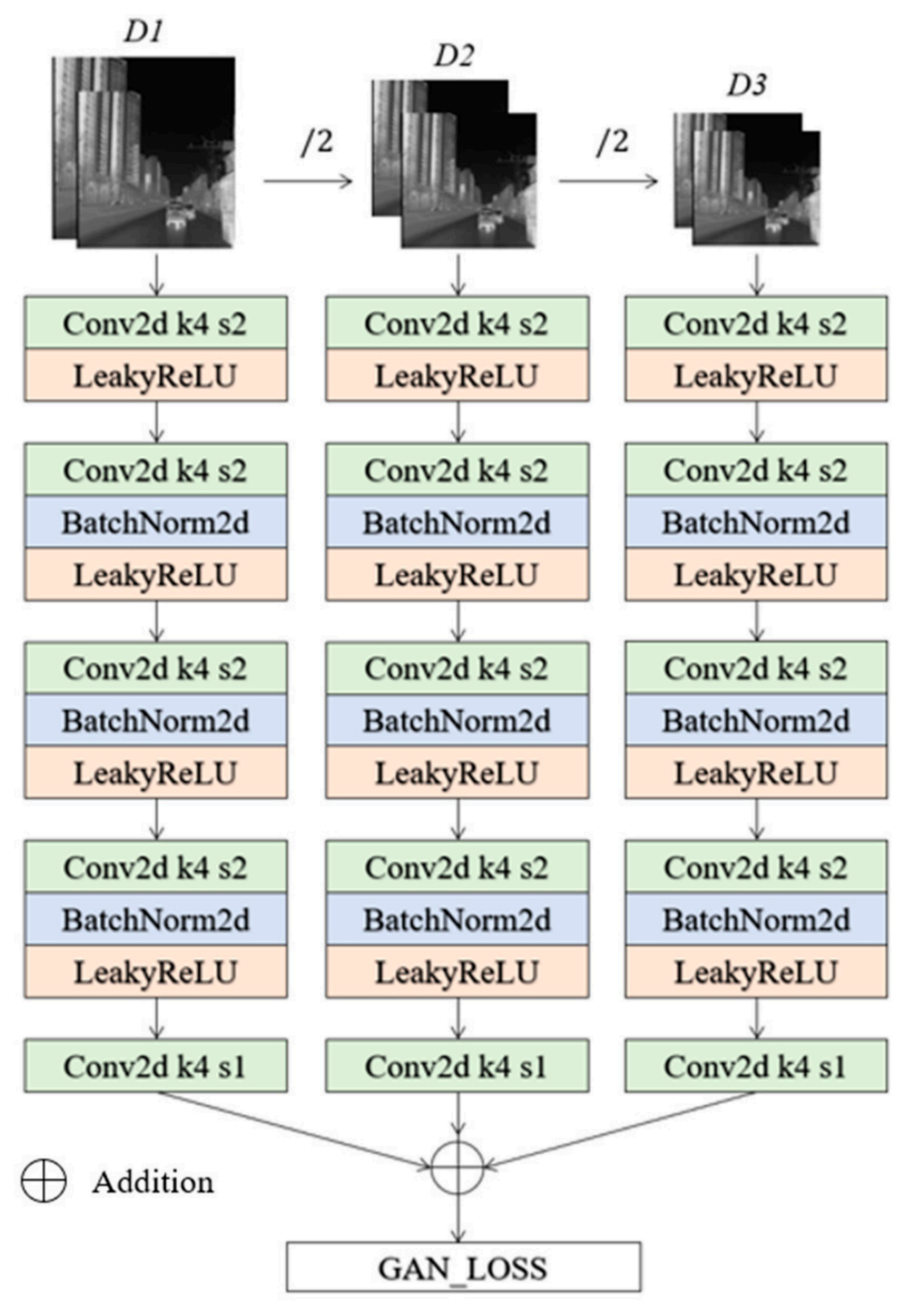
Diagram of multi-scale discriminator. Three branches receive images at different resolutions to extract local texture and global structural features simultaneously, and the fused outputs are used to calculate the adversarial loss to constrain the quality of generated infrared images.

As illustrated in Figure 3, the proposed framework integrates three discriminators with different scales, denoted as D1, D2, and D3. Identical architectures are adopted by all three discriminators. The input of D2 is obtained by 2× downsampling on the input of D1, and the input of D3 is generated by another 2× downsampling on the input of D2. Each discriminator consists of five convolutional layers with 4 × 4 kernels. A stride of 2 is adopted in the first four convolutional layers to simultaneously reduce channel dimensions and spatial resolution, while the last layer only adjusts channel numbers for feature aggregation and outputs one-dimensional feature values corresponding to each scale. Despite their identical network structures, the three discriminators receive inputs of different resolutions. Under the same receptive field size with equivalent parameter overhead, they can capture distinct feature patterns of infrared images. D1 takes high-resolution images as input and concentrates on local fine-grained cues such as textures and edges. In contrast, D2 and D3 process low-resolution downsampled images to capture global structural information including scene layout and object shapes. Consequently, the multi-scale discriminator comprehensively learns both local and global image features, which effectively guides the generator to produce high-fidelity infrared images.

### 2.3. Loss Function Design

#### 2.3.1. Adversarial Loss

The generator loss is derived from the classification of generated images as fake samples by the discriminator, while the discriminator loss is induced by the misclassification of input images. An adversarial scheme is therefore established between the two networks. Conventional adversarial loss adopts *JS* divergence and *KL* divergence as measurement metrics, as formulated in Equation (2), where *p* denotes the real data distribution, and *q* represents the generated distribution. Kullback–Leibler (*KL*) divergence is adopted to measure the discrepancy between these two distributions. When the distributions *p* and *q* are either fully overlapped or mutually disjoint, the loss value converges to log(2), entering a saturation zone where the loss function yields no effective gradient signals. To address this issue, the least-squares (LS) loss function is introduced in this paper. The overall LS-GAN adversarial loss is defined in Equation (3), which consists of two branch terms. The LS loss for infrared image generator *G* and visible-image generator *F* are given in Equations (4) and (5), respectively. Therefore, the saturation problem is eliminated, and the distance between *fake_ir_* and the decision boundary of the discriminator is penalized. Early training stagnation of the generator is avoided, continuous optimization is facilitated, and the quality of *fake_ir_* is ultimately improved.

(2) $$\mathrm{JS}(p∥q)=\frac{1}{2}\mathrm{KL}\left(p∥\frac{p+q}{2}\right)+\frac{1}{2}\mathrm{KL}\left(q∥\frac{p+q}{2}\right)$$

*L*_*GAN*_all__ = *L_GAN_*(*G*, *D_Y_*, *X*, *Y*) + *L_GAN_*(*F*, *D_X_*, *Y*, *X*) (3)

(4) $$L_{\mathrm{GAN}}(G,D_{Y},X,Y)=\frac{1}{2}E_{x}[{\left(D_{Y}(G(x))-1\right)}^{2}]+\frac{1}{2}E_{y}[{\left(D_{Y}(y)-1\right)}^{2}]+\frac{1}{2}E_{x}[{\left(D_{Y}(G(x))\right)}^{2}]$$

(5) $$L_{\mathrm{GAN}}(F,D_{X},Y,X)=\frac{1}{2}E_{y}\left[{\left(D_{X}(F(y))-1\right)}^{2}\right]+\frac{1}{2}E_{x}\left[{\left(D_{X}(x)-1\right)}^{2}\right]+\frac{1}{2}E_{y}\left[{\left(D_{X}(F(y))\right)}^{2}\right]$$

where *x* denotes visible images, *y* denotes infrared images, *X* represents the visible domain, *Y* represents the infrared domain, *G* is the infrared image generator, *D_Y_* is the infrared domain discriminator, *F* is the visible-image generator, and *D_X_* is the visible-domain discriminator. *E_x_*[⋅] denotes the mathematical expectation of sample *x* over the visible-light domain X, and *E_y_*[⋅] refers to the expectation of sample *y* over the infrared domain *Y*. The loss term $E_{x}[{\left(D_{Y}(G(x))-1\right)}^{2}]$ is adopted to optimize the generator *G*. It aims to encourage the discriminator to classify the synthesized infrared image (*fake_ir_*) as real (label = 1), and the least-squares form imposes penalty on errors that deviate from the ground-truth label. The term $E_{y}[{\left(D_{Y}(y)-1\right)}^{2}]$ is used to optimize the discriminator *D_Y_*, which guides the network to classify the real infrared image (*real_ir_*) as authentic. The term $E_{x}[{\left(D_{Y}(G(x))\right)}^{2}]$ serves for the optimization of discriminator *D_Y_*, so that the synthesized infrared image *fake_ir_* is identified as fake.

#### 2.3.2. Multi-Layer Contrastive Loss

**Figure 4:**
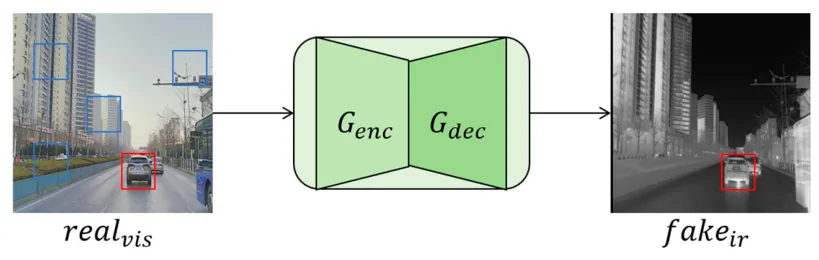
Positive or negative samples in contrastive learning.

For the visible-to-infrared cross-domain generation task, the *real_ir_* and *fake_ir_* are required to maintain consistent semantic and spatial information, which corresponds to the inherent shared attributes between the two domains. To satisfy this constraint, a multi-layer contrastive loss is incorporated into the proposed framework. The core objective of the multi-layer contrastive loss framework is to realize feature matching between each query sample and its corresponding positive sample, while suppressing interference caused by irrelevant negative samples. This mechanism preserves shared cross-domain features during image translation. As shown in Figure 4, the vehicle region (red box) in *fake_ir_* is taken as the query sample. The vehicle region enclosed by the red box in is served as *real_vis_* the positive sample with identical semantic and spatial content. All arbitrary regions outside this matching area (blue boxes) are treated as negative samples.

The query sample, positive sample, and N negative samples are mapped into P-dimensional vectors, denoted as **v** ∈ ℝ*^P^*, **v**^−^ ∈ ℝ*^N^*^×*P*^ and **v**^+^ ∈ ℝ*^P^*. All P-dimensional vectors are normalized by the L2 norm, which transforms the feature matching task into a classification problem to calculate the probability of matching the positive sample. Mathematically, this objective is formulated with cross-entropy loss as follows:

(6) $$\Gamma \left(v,v^{-},v^{+}\right)=-\log\left[\frac{\exp\left(\mathrm{sim}(v,v^{+})/\tau \right)}{\exp\left(\mathrm{sim}(v,v^{+})/\tau \right)+\sum_{n=1}^{N}\exp\left(\mathrm{sim}(v,v^{-})/\tau \right)}\right]$$

where $\mathrm{sim}(v,u)=\frac{v^{T}u}{\mathrm{\|u\| \|v\|}}$ denotes the cosine similarity between *v* and *u*. τ is the distance scaling factor between the query sample and other samples, and we adopt 0.07 as its default value in this work.

*G_enc_* and *H*_vis_ are employed to extract features from the visible domain, while *F_enc_* and Hir are utilized for feature extraction in the infrared domain. To effectively learn and capture the variability within the two domains, independent weights are adopted for the two feature extraction networks. For the visible domain, L-layer features extracted by *G_enc_* are fed into *H*_vis_ and aggregated into one-dimensional features, which are formulated as:

(7) $${\left\{Z_{l}\right\}}_{L}={\left\{H_{\mathrm{vis}}^{l}(G_{\mathrm{enc}}^{l}(x))\right\}}_{L}$$

where $G_{\mathrm{enc}}^{l}(x)$ denotes the output of the l-th layer. The spatial position of the query sample is denoted as *s* ∈ {1,…,*S*_1_}, where *S_l_* stands for the total number of spatial positions at layer l.

For each query, the feature at the query position is selected as the positive sample, denoted by $Z_{l}^{s}\in {\mathbb{R}}^{C_{l}}$, where Cl represents the number of channels of this layer. The remaining features are treated as negative samples, written as $Z_{l}^{S_{l}\setminus s}\in {\mathbb{R}}^{(S_{l}-1)\times C_{l}}$, which correspond to features from all spatial positions excluding the query location. Similarly, feature extraction performed on *fake_ir_* yields a new set of features, formulated as:

(8) $${\left\{{\hat{Z}}_{l}\right\}}_{L}={\left\{H_{\mathrm{ir}}^{l}(F_{\mathrm{enc}}^{l}(G(x)))\right\}}_{L}$$

Thus, the multi-layer contrastive loss function for visible-to-infrared generation can be formulated as:

(9) $$L_{{\mathrm{PatchNCE}}_{X}}(G,H_{\mathrm{vis}},H_{\mathrm{ir}},X)=E_{x\sim X}\sum_{l=1}^{L}\sum_{s=1}^{S_{l}}l\left({\hat{Z}}_{l}^{s},Z_{l}^{s},Z_{l}^{S_{l}\setminus s}\right)$$

Benefiting from the symmetric network architecture, we introduce analogous loss terms for the reverse mapping, i.e., infrared-to-visible translation, as illustrated in Equations (10)–(13):

(10) $${\left\{Z_{l}\right\}}_{L}={\left\{H_{\mathrm{ir}}^{l}(F_{\mathrm{enc}}^{l}(y))\right\}}_{L}$$

(11) $${\left\{{\hat{Z}}_{l}\right\}}_{L}={\left\{H_{\mathrm{vis}}^{l}(G_{\mathrm{enc}}^{l}(F(y)))\right\}}_{L}$$

(12) $$L_{{\mathrm{PatchNCE}}_{Y}}(F,H_{\mathrm{ir}},H_{\mathrm{vis}},Y)=E_{y\sim Y}\sum_{l=1}^{L}\sum_{s=1}^{S_{l}}l\left({\hat{Z}}_{l}^{s},Z_{l}^{s},Z_{l}^{S_{l}\setminus s}\right)$$

*L*_*P*atch*NCE*_all__ = *L*_*P*atch*NCE_X_*_ + *L*_*P*atch*NCE_Y_*_ (13)

#### 2.3.3. Composite Loss Components and Total Objective

(1) Unidirectional Cycle-Consistency Loss

A unidirectional cycle-consistency loss function is devised to regularize the generator during source-to-target domain translation. It constrains the generator to preserve image details and recover the source-domain image to the greatest extent in the subsequent processing stage. This loss guarantees that the generator can not only synthesize visually realistic target-domain images but also retain critical source-domain information throughout translation, so as to establish an effective mapping between the source domain and the target domain. The loss is formulated as:

*L_cyc_* = 𝔼*_x_*_~*X*_‖*F*(*G*(*x*)) − *x*‖_1_ (14)

(2) Identity Loss

Deviations occur in the internal network mapping during infrared image generation. Such deviations may alter color details of the input image and lead to undesired shifts in brightness information within the infrared outputs. To tackle this issue, an identity loss function is introduced to correct the bias of the generator during training and encourage the generator to produce features corresponding to more realistic infrared images. As shown in Equation (15), the L1 loss function is adopted to calculate the identity loss.

*L_ide_* = 𝔼*_x_*_~*X*_‖*F*(*x*) − *x*‖_1_ + 𝔼*_y_*_~*Y*_‖*G*(*y*) − *y*‖_1_ (15)

(3) Total Loss Function

In our method, we clearly distinguish loss terms for generators and discriminators. Only the least-squares adversarial loss (LS-GAN loss) is used to update the parameters of the multi-scale discriminators *D_ir_* and *D_vis_*. The multi-layer contrastive loss, unidirectional cycle-consistency loss, and identity feature loss are only back-propagated to update the generators, and these loss terms do not contribute to discriminator parameter optimization. For the two generators (*G* for visible-to-infrared translation and *F* for *λ* infrared-to-visible translation), the full optimization objective combines four weighted components: least-squares adversarial loss, multi-layer contrastive loss, unidirectional cycle-consistency loss, and identity feature loss. During training, we adopt an alternating optimization strategy: discriminators are updated first, followed by the joint update of two generators under the weighted composite loss. The overall objective functions are formulated as:

(16) $$L_{G}=\lambda_{\mathrm{adv}}L_{\mathrm{GAN}}^{G}+\lambda_{\mathrm{nce}}L_{\mathrm{nce}}+\lambda_{\mathrm{cyc}}L_{\mathrm{cyc}}+\lambda_{\mathrm{ide}}L_{\mathrm{ide}}$$

(17) $$L_{D}=L_{\mathrm{GAN}}^{D}$$

where *λ_adv_* = 1, *λ_nce_* = 5, *λ_cyc_* = 20, and *λ_ide_* = 10 are the fixed weighting coefficients adopted in our experiments.

The aforementioned loss terms impose impacts of varying magnitudes on the simulated infrared image generation performance. By assigning weights to individual loss components, the overall loss function of CVIGAN is constructed, as presented in Equation (18). Extensive experiments reveal that the model achieves optimal performance under the parameter combination *λ_GAN_* = 1, *λ_NCE_* = 5, *λ_ide_* =10, and *λ_cyc_* =20 when the total training epoch is set to 200. Here, *λ_GAN_*, *λ_NCE_*, *λ_ide_* and *λ_cyc_* denote weight coefficients. The formulation of the total loss function is given as:

(18) $$L_{\mathrm{total}}=\lambda_{\mathrm{GAN}}L_{{\mathrm{GAN}}_{\mathrm{all}}}+\lambda_{\mathrm{NCE}}L_{{\mathrm{PatchNCE}}_{\mathrm{all}}}+\lambda_{\mathrm{ide}}L_{\mathrm{ide}}+\lambda_{\mathrm{cyc}}L_{\mathrm{cyc}}$$

## 3. Experiments and Results

To verify the effectiveness of the proposed method, extensive experiments are conducted to evaluate the performance of the proposed infrared image generation algorithm.

### 3.1. Datasets

In this paper, experiments are carried out on the M3FD [23] and AVIID3 [24] datasets and the self-built SCHUAN dataset. A combined dataset with diverse shooting viewpoints and scenarios is constructed to validate the model performance and generalization capability for objects under varying viewpoints and scales.

**Figure 5:**
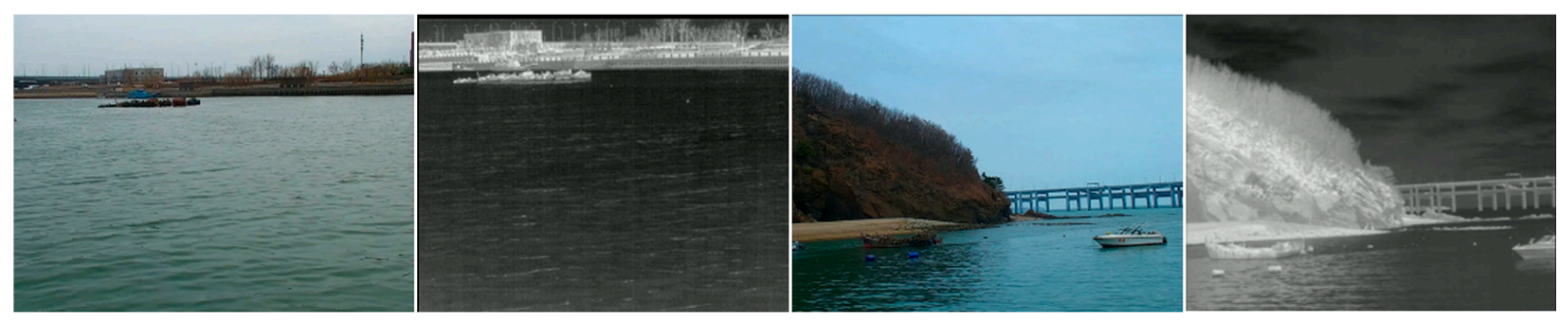
Samples from the SCHUAN Dataset.

1. AVIID3 Dataset: This dataset consists of 1280 pairs of visible-infrared images. The training set contains 1024 visible images and 1024 infrared images. To maintain the unsupervised learning setup, images are fed into the network in a random order during training. The remaining 109 pairs form the test set. Data are captured by UAVs at three different altitudes, covering complex backgrounds such as roads, river bridges, parking lots, and streets in residential communities. This dataset exhibits rich diversity and features relatively small objects compared with other datasets, making it suitable for visible-to-infrared translation tasks of small targets.
2. M3FD Dataset: It is a multispectral dataset captured in highway scenarios. The dataset covers abundant scenes with visible and infrared images of vehicles, buildings, and other objects. A total of 1133 high-quality image pairs are selected as experimental samples.
3. SCHUAN Dataset: Maritime visible-infrared data are collected using the FLIR M300 dual-spectrum camera. As shown in Figure 5, the visible and infrared cameras differ in installation position, field of view, and frame rate. These discrepancies lead to inconsistent imaging between visible and infrared modalities, forming an unpaired training setting to evaluate the proposed method. The training set contains 664 unpaired visible images and 664 unpaired infrared images. To provide paired samples for testing, another 45 groups of images outside the training set are selected, and semi-manual registration is performed between visible and infrared images. Owing to the inherent parallax between cameras, residual sub-pixel misalignment exists after registration, which limits the upper bound of pixel-dependent metrics PSNR and SSIM. Therefore, LPIPS, FID, and KID are used as primary metrics, while PSNR and SSIM serve as secondary references for SCHUAN, and structural-consistency conclusions will not be drawn merely from PSNR/SSIM.
4. Campus Dataset

We collected 20 visible images on the Dalian Maritime University campus for out-of-distribution generalization and downstream object detection evaluation. These images cover typical campus road scenes and mainly contain vehicle targets. The YOLOv5 detector is pre-trained on the labeled M3FD dataset, and no manual annotations are required for these campus images.

### 3.2. Experimental Setup

#### 3.2.1. Experimental Platform

The training platform configuration for all deep learning experiments in this work is listed as follows: the graphics card is a GeForce RTX 2080Ti with 11 GB of video memory. The deep learning framework is PyTorch 1.6.0, the Python version is 3.7, and the operating system is Windows 10.

#### 3.2.2. Experimental Hyperparameters

For ablation studies and comparative experiments, the batch size is set to 1 for all experiments, and the Adam optimizer is adopted. The network of the proposed method is trained for 200 epochs to guarantee convergence. The learning rate of the generator is fixed at 0.0002 for the first 100 epochs and linearly decays to zero over the subsequent 100 epochs. The learning rate of the discriminator is fixed at 0.000002 for the first 100 epochs and linearly decays to zero over the remaining 100 epochs. The optimal model performance is achieved with the weight coefficients *λ_GAN_* = 1, *λ_NCE_* = 5, *λ_ide_* =10, and *λ_cyc_* =20. CycleGAN is trained for 200 epochs, while GLANET, DCLGAN, SimDCL, CUT, UNSB, and DR-AVIT are trained for 400 epochs.

To ensure fair comparison among all competing approaches, we adopt the following baseline tuning, validation, and checkpoint-selection protocol.

For each baseline method, we perform a limited hyper-parameter sweep on our target datasets (M3FD, AVIID3, SCHUAN). We search over core hyper-parameters including learning rate and loss weight coefficients within the typical ranges suggested in each original paper, while keeping other network architectural settings unchanged. The hyper-parameter combination achieving the best overall performance on the validation split is retained for subsequent test-set evaluation.

We randomly split each dataset into training set, validation set, and test set. The test set is strictly held-out and never seen during hyper-parameter tuning or model training. It is worth noting that training epochs alone cannot represent equal computational budget, since per-epoch computational complexity varies across different network architectures. Accordingly, rather than simply employing the weights from the final training epoch, we select the model checkpoint that yields the best comprehensive metric (average of SSIM, PSNR and LPIPS) on the validation set for testing and comparison, regardless of whether this checkpoint appears at epoch-200, epoch-400 or other iterations. All models are evaluated on the identical held-out test set under consistent inference configurations.

#### 3.2.3. Evaluation Metrics

Two groups of evaluation metrics are introduced in this paper to comprehensively evaluate the model performance. Specifically, one group evaluates the qualitative and quantitative quality of *faker_ir_*, while the other focuses on the downstream object detection task to verify the practical application capability of *faker_ir_*.

1. Image Generation;

- Structural Similarity Index Measure (SSIM) [25]: The similarity of luminance, contrast, and structural information is quantified between generated images and real reference images. Its value ranges from 0 to 1. A value closer to 1 indicates higher structural consistency and lower distortion; a value of 0 means no similarity, while negative values represent completely opposite structures;
- Peak Signal-to-Noise Ratio (PSNR) [26]: The pixel-level discrepancy is measured between generated images and reference images. Higher PSNR values denote greater similarity between two images;
- Learned Perceptual Image Patch Similarity (LPIPS) [27]: It is a deep-learning-based metric to evaluate image similarity. Features are extracted via a pre-trained deep neural network, and the distance in feature space is calculated to measure the similarity. LPIPS is only used for evaluation and is not involved in model training;
- Fréchet Inception Distance (FID) [28]: Image features are extracted by the Inception network to compute the distance between the feature distribution of generated images and that of real samples. A lower FID implies that the overall style and content distribution of synthesized images are closer to real data without severe blurring, distortion, or artifacts. A higher FID indicates a large distribution gap between generated and real images;

2. Object Detection;

The detailed downstream detection metrics are elaborated as follows.

- Recall: Recall is defined as the ratio of correctly detected true positive samples to all ground-truth samples, which is adopted to quantify the missing detection rate;
- Precision: *Precision* is defined as Equation (19), where *TP* (true positive) denotes a predicted bounding box that matches a ground-truth box with IoU greater than the predefined threshold and confidence score higher than the confidence threshold. *FP* (false positive) represents a predicted box that fails to meet the above IoU matching condition.
  *Precision* = *TP*/(*TP* + *FP*) (19)
- mean Average Precision (mAP): mAP is a core metric for evaluating the overall performance of multi-class object detection algorithms. The value ranges from 0 to 1, where a higher value indicates a better detection performance. Specifically, mAP50 denotes the mAP calculated under an IoU threshold of 0.5, which is widely used to evaluate detection capability under loose localization constraints. Furthermore, mAP50@95 averages the mAP values computed at ten IoU thresholds spanning from 0.5 to 0.95 with a step size of 0.05. Thus, mAP50@95 imposes stricter requirements on the localization accuracy of bounding boxes and comprehensively reflects algorithm performance under diverse localization precision requirements.

### 3.3. Experimental Results

#### 3.3.1. Comparative Experiments

**Figure 6:**
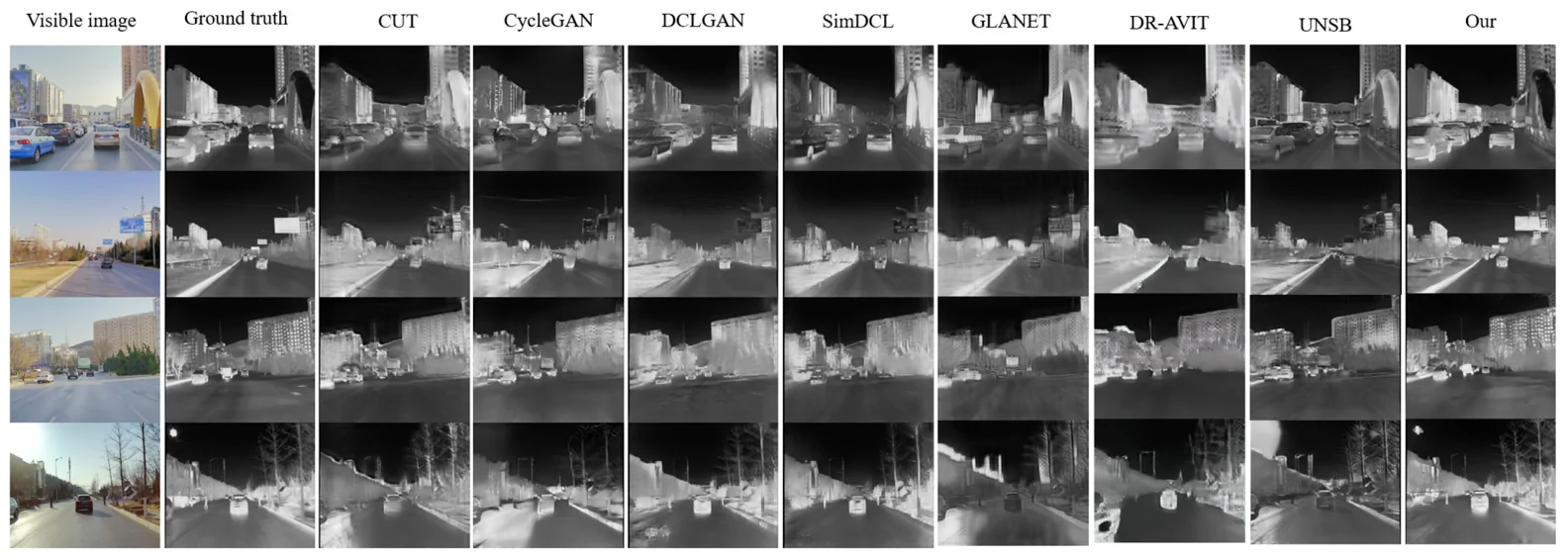
Algorithm comparison on the M3FD dataset. The proposed approach generates infrared images with more accurate thermal characteristics and clearer object details compared with other competing algorithms.

The superiority of the proposed CVIGAN for infrared image generation is verified by performance comparison with seven representative image translation networks, including CycleGAN, CUT, GLANET [29], DCLGAN, SimDCL [30], UNSB, and DR-AVIT [31]. Hyperparameter configurations reported in the original papers are adopted for all the competing methods, and comparative experiments are conducted on two distinct datasets. To comprehensively compare the performance gaps among different algorithms, we evaluate the infrared image generation results from two perspectives: objective quantitative analysis and subjective visual assessment. Qualitative results on the M3FD dataset are demonstrated in Figure 6. It can be clearly observed that CycleGAN, GLANET, and CUT fail to learn plausible infrared visual patterns of objects. In real infrared images, moving vehicles tend to present brighter grayscale regions, and wheels often show stronger pixel intensity than the vehicle body. In the fourth row of Figure 6, comparisons between infrared outputs generated by the three methods and ground-truth images show that the generated vehicles exhibit visually unreasonable thermal patterns. Partial thermal features of objects are captured by DCLGAN and SimDCL, yet structural information from visible scenes is poorly preserved. As shown in the third row, distorted window structures of buildings and blurry scene contents are observed. Disentangled representations and geometric constraints are leveraged by DR-AVIT to preserve scene features and produce diverse infrared outputs. Nevertheless, local details are lost owing to global style disentanglement. As displayed in the second row of Figure 6, infrared signatures of distant small vehicle targets cannot be accurately generated by DR-AVIT. Regularization and contrast learning are integrated in UNSB, and multi-round iterations are adopted to retain details and mitigate information loss during cross-domain translation. However, implausible infrared visual patterns are generated for vehicles.

Furthermore, the images produced by the proposed method achieve higher consistency with *real_ir_*, compared with those yielded by other competing methods. As shown in Table 1, our method achieves obvious improvements in PSNR and SSIM, which verifies its superiority in pixel-level fidelity and structural preservation. For perception-oriented metrics including LPIPS and FID, the proposed approach achieves the lowest LPIPS value among all competing methods, while obtaining a competitive FID score that is marginally higher than the optimal value from UNSB. It demonstrates that the distributions of fake_ir_ are well aligned with those of real *real_ir_* in the perceptual space, and improved visual naturalness as well as semantic consistency can be attained.

**Figure 7:**
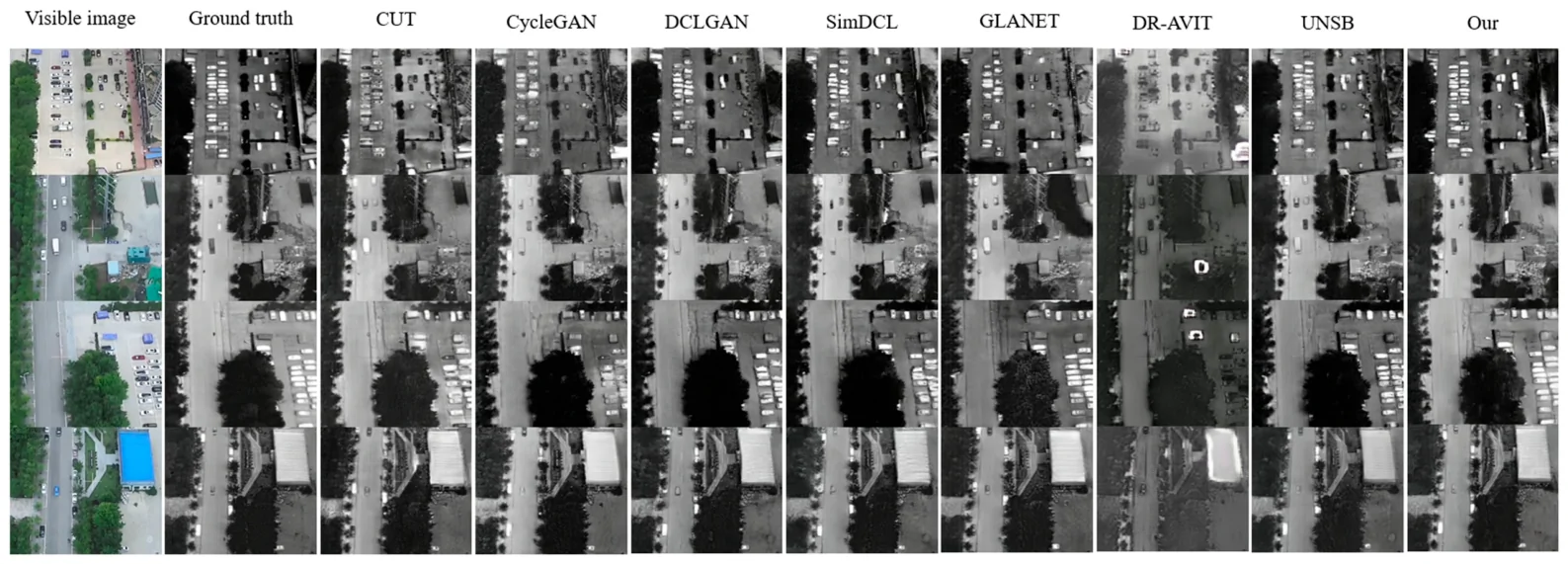
Algorithm comparison on the AVIID3 dataset. Our method achieves more accurate infrared thermal mapping and preserves fine-grained details for small objects compared with competing approaches.

To further verify the effectiveness of the proposed method, the AVIID3 dataset with more complex scenarios and smaller target objects is adopted for systematic evaluation of different algorithms. As shown in Figure 7, accurate feature mapping for small infrared targets cannot be realized by methods including CUT, CycleGAN, and DR-AVIT, and the synthesized vehicles fail to present plausible infrared visual patterns. Comparisons with ground-truth infrared images reveal that partial scene information loss is suffered by the UNSB method during translation. Although scene features from visible images can be retained to a certain extent by SimDCL and DCL, the boundaries between generated objects are blurred. Especially in densely distributed vehicle regions, gap details among vehicles cannot be accurately reconstructed. Taking all visual results into consideration, our method achieves prominent advantages in both detail restoration and infrared visual pattern generation.

As listed in Table 2, our approach outperforms all competing algorithms by a notable margin on all objective metrics over the AVIID3 dataset. Specifically, our method achieves an SSIM of 0.5710, approximately 4.9% higher than the suboptimal SimDCL. The PSNR reaches 21.72 dB, surpassing SimDCL by nearly 1.06 dB. The lowest LPIPS value of 0.1700 is obtained, which is reduced by 15.0% compared with SimDCL. Meanwhile, the FID score is 58.48, which is improved by 5.4% relative to SimDCL. These quantitative results sufficiently demonstrate that the proposed method achieves remarkable advantages in preserving image structural integrity, boosting signal-to-noise performance, improving perceptual visual quality, and enhancing the authenticity of feature distributions. Meanwhile, the performance of each network varies across different scenarios. For visible-to-infrared translation tasks, our method achieves state-of-the-art performance under diverse scene conditions.

**Figure 8:**
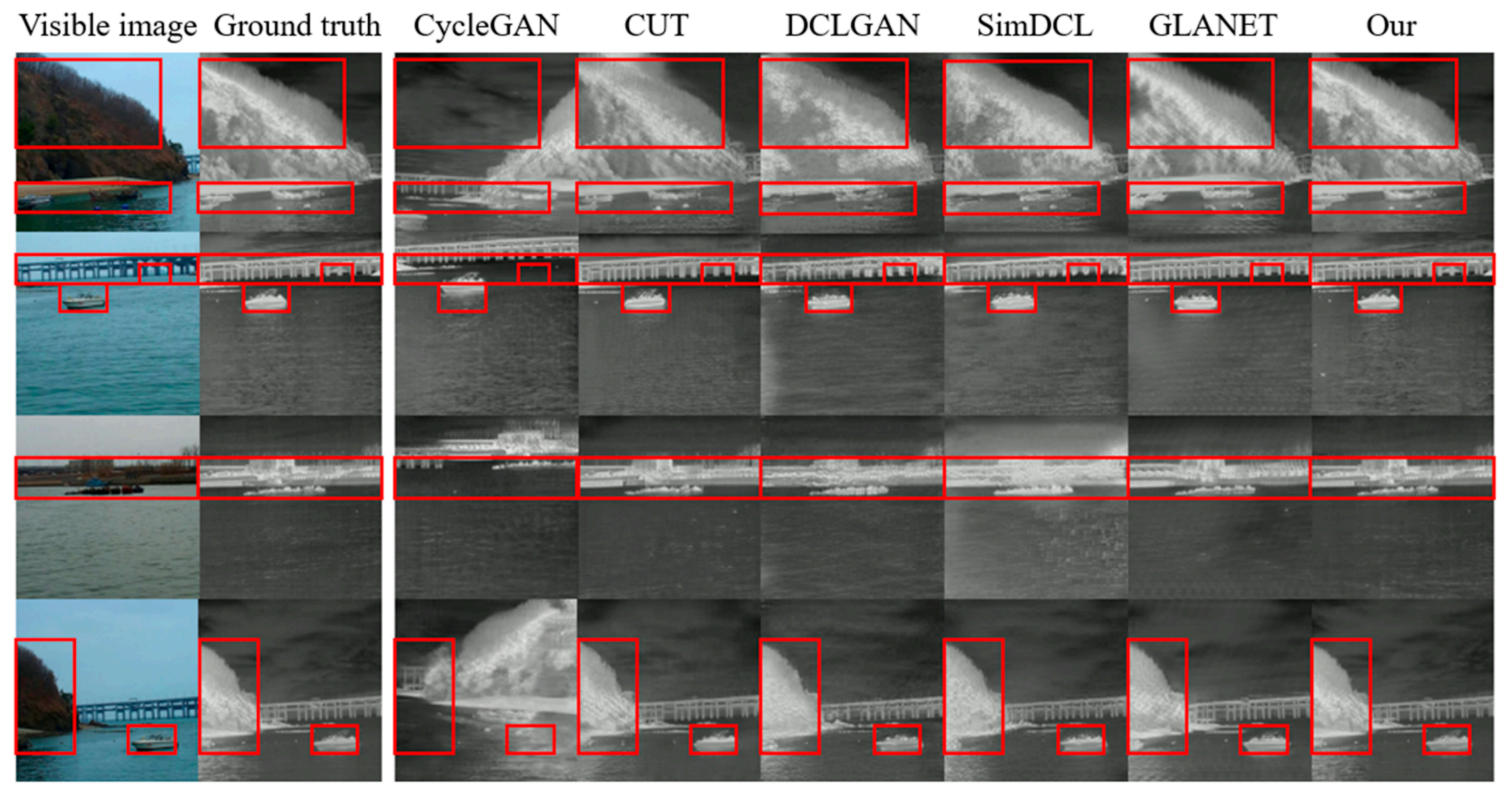
Comparison of different algorithms on the SCHUAN dataset. Highlighted by red boxes, our method effectively avoids object displacement and grid artifacts, yielding infrared outputs that are more consistent with ground-truth thermal features.

To verify the applicability of the proposed method in maritime scenarios, experiments are conducted on the SCHUAN dataset. Compared with *real_ir_*, CycleGAN-produced *fake_ir_* exhibit spatial dislocation and fail to properly restore object spatial positions from *real_vis_*. As shown in the fourth row of Figure 8, grid artifacts are produced over reef areas by GLANET and CUT, resulting in blurry visual outputs. As displayed in the first row of Figure 8, ships disappear within the regions marked by red boxes in the results of DCLGAN and SimDCL.

Table 3 lists quantitative results on the SCHUAN dataset. CVIGAN outperforms all baselines on all metrics. LPIPS and FID are adopted as primary metrics for this dataset, and our method achieves the optimal LPIPS and FID. PSNR and SSIM serve as secondary references due to residual registration misalignment, and our method also achieves the best relative results on these two metrics.

By enhancing feature extraction and reconstruction, together with constraints imposed by the discriminator and loss functions, the proposed method enables the *fake_ir_* to capture the thermal signatures of scenes, capturing visually plausible scene thermal patterns while preserving fine textures and edge information from *real_ir_*. Grid artifacts and spatial dislocation are avoided, yielding outputs much closer to *real_ir_*.

#### 3.3.2. Ablation Experiments

**Figure 9:**
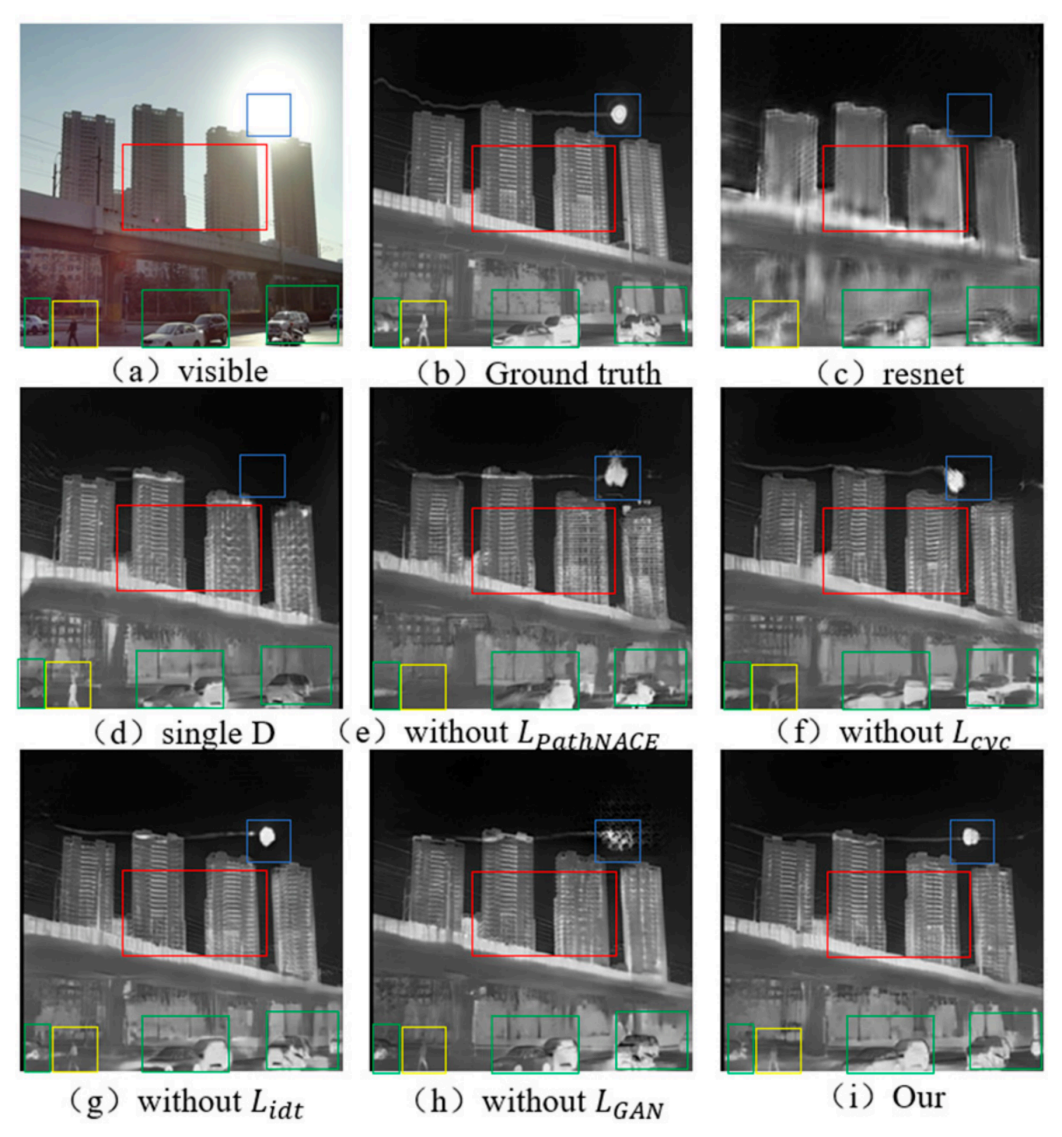
Ablation experiment data graph on the M3FD dataset. The proposed full model (i) retains complete object structures and thermal details within the marked boxes, demonstrating the necessity of each designed component. The red, blue, green, and yellow boxes denote high-rise buildings, sun region, vehicles, and pedestrians, respectively.

Four loss functions, including GAN loss, multi-layer contrastive loss, unidirectional cycle-consistency loss, and identity loss, are integrated to train the network model. To verify the effectiveness of each loss function as well as the designed generator and discriminator, ablation experiments are carried out. The experimental results are presented in Table 4 and Figure 9. In these experiments, we sequentially replace the customized generator with a residual network, substitute the designed multi-scale discriminator with a single discriminator, remove multi-layer contrastive loss, identity loss, and unidirectional cycle-consistency loss, and adopt JS loss instead of LS loss as the adversarial loss. All other network components remain unchanged during each comparison.

As indicated by the yellow boxes in Figure 9, result (i) produced by the proposed method preserves the spatial positions of objects well, and the disappearance of human targets observed in subfigure (e) is avoided. As marked by green boxes, our method successfully captures vehicle regions with distinct edge details and plausible infrared visual patterns. In the regions highlighted by red boxes, feature details of buildings are better retained by the proposed method, and clearer *faker_ir_* are yielded. As summarized in Table 4, performance degradation is observed when any component is removed, demonstrating that each component contributes positively to structural preservation of the *faker_ir_*. The generator imposes the most significant impact on the whole network, which further validates the effectiveness of the proposed algorithm.

#### 3.3.3. Scene Generalization Experiments

**Figure 10:**
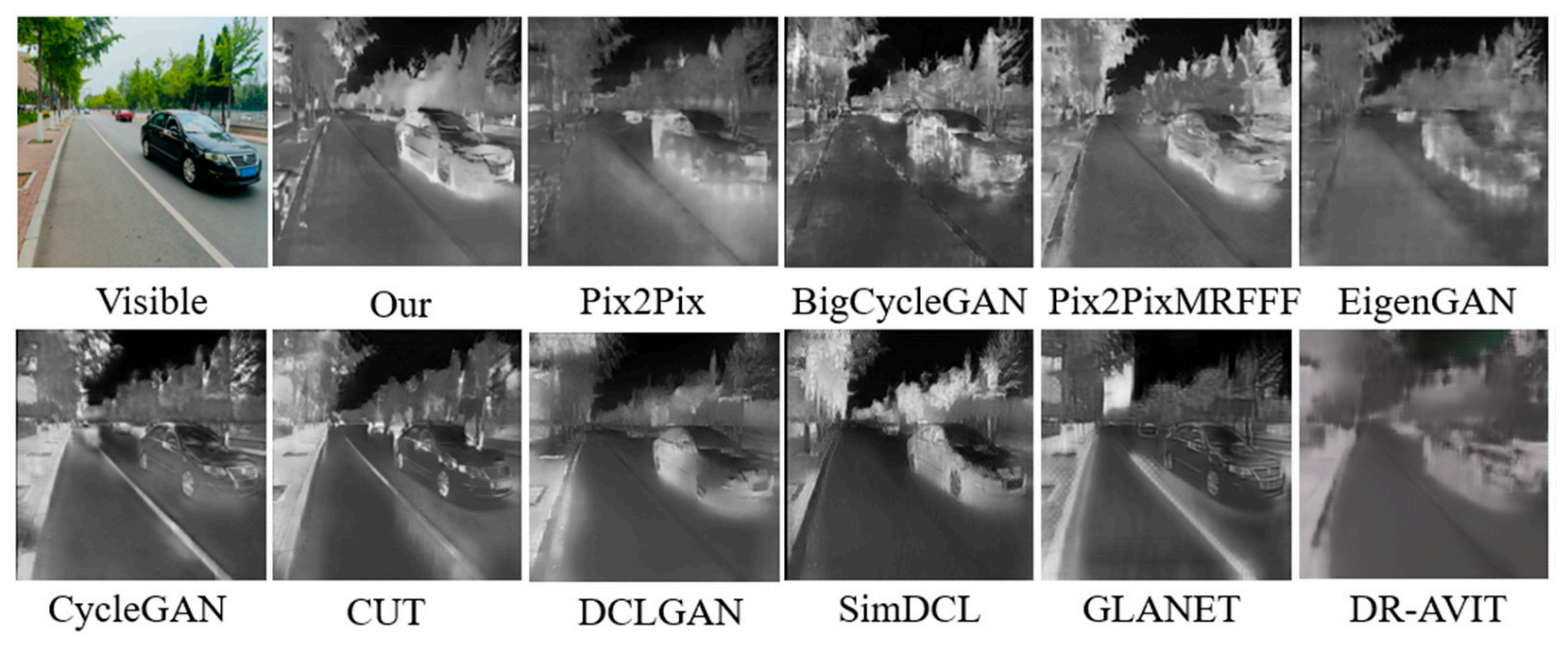
Infrared images generated by different algorithms under cross-dataset scenarios.

To comprehensively evaluate the out-of-dataset generalization capability of CVIGAN, visible images are captured on the campus of Dalian Maritime University for comparative tests using all aforementioned methods. All image-translation models are pre-trained on the M3FD dataset, and the campus images are only used for testing without participating in model training. This newly collected campus dataset is also adopted for downstream quantitative object-detection evaluation. As shown in Figure 10, compared with ground-truth infrared images, real infrared samples, among the image translation methods trained on paired data, Pix2Pix and Big-CycleGAN [32] produce outputs with blurry object boundaries. Pix2Pix-MRFFF [33] and EigenGAN [34] suffer from small-target disappearance; distant small vehicle targets fail to be mapped correctly. For infrared generation methods designed for unpaired training data, CycleGAN, CUT, and GLANET fail to reconstruct reasonable scene infrared visual patterns. Although DCLGAN, SimDCL, and DR-AVIT are capable of generating plausible infrared appearances, their outputs are over-smoothed, leading to detail loss and inaccurate preservation of object positions. The proposed CVIGAN achieves better performance than existing methods on this newly collected dataset. Particularly in scene detail restoration and thermal signature reconstruction, it effectively recovers fine details while generating visually reasonable thermal characteristics for target objects.

**Figure 11:**
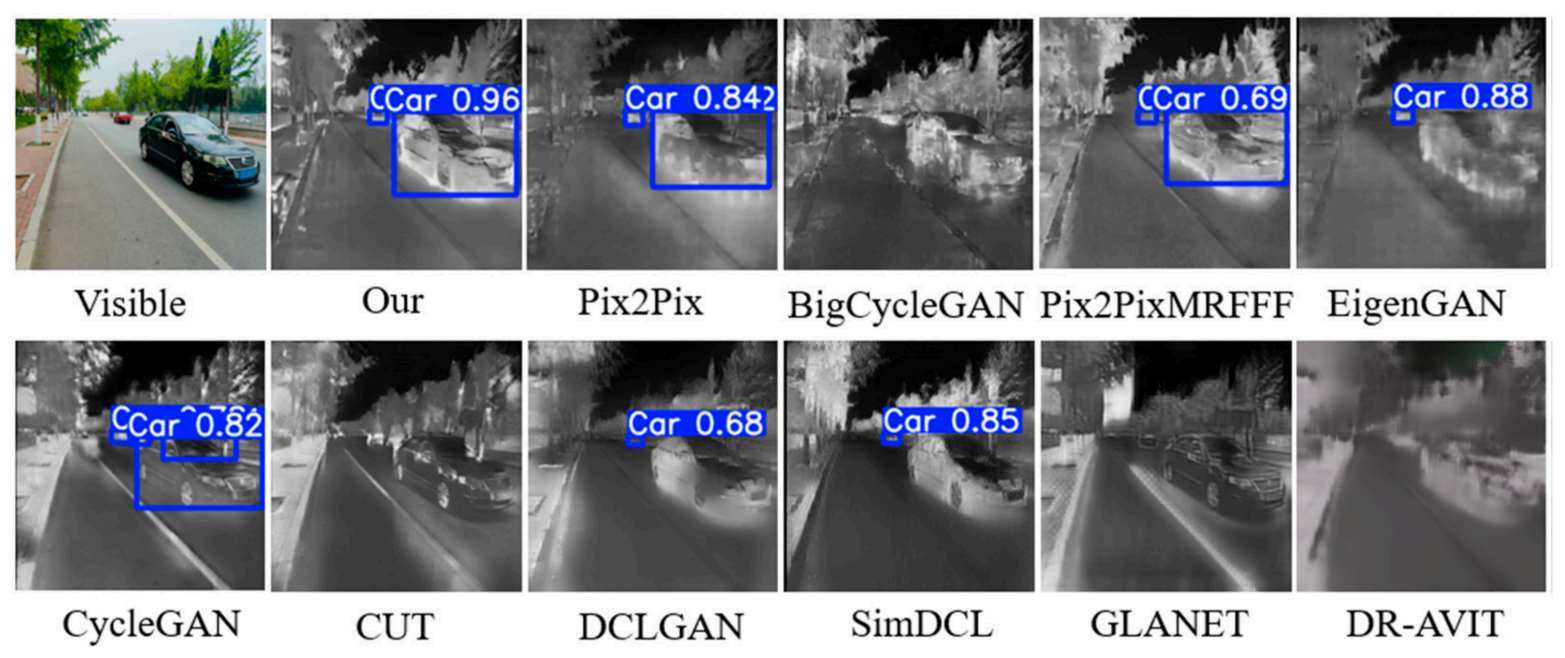
Comparison of detection results on infrared images.

Meanwhile, the YOLOv5 [35] network is trained on the labeled infrared samples from the M3FD dataset. Infrared images generated by different comparison algorithms from campus visible inputs are fed into this fixed YOLOv5 model for detection evaluation to verify the effectiveness of the proposed method in downstream tasks. During inference, YOLOv5s is adopted with a confidence threshold of 0.25 and an IoU threshold of 0.45 for non-maximum suppression. As shown in Figure 11, our method can accurately locate vehicles, achieving the highest confidence score of 0.96. Nevertheless, the smallest distant vehicles cannot be identified precisely due to the limitation of image resolution.

As listed in Table 5, comparative results reveal that other competing models suffer from unbalanced performance trade-offs. SimDCL achieves a relatively high precision of 0.782, but only obtains a recall of 0.25. Pix2Pix reaches a recall of 0.625, while its mAP@50 is merely 0.522. Optimal results in terms of precision, recall, and mAP are acquired by the proposed CVIGAN. It demonstrates that *faker_ir_* possess high visual fidelity and can provide effective feature information for downstream object detection tasks, which verifies the technical effectiveness and practical value of CVIGAN in the field of infrared image generation.

#### 3.3.4. Cross-Scale Generalization Experiments

Restricted by the computing and storage resources of the experimental platform, it is difficult to conduct large-scale iterative training directly using high-resolution raw infrared images such as 640 × 512 and 1024 × 768 during model training. Accordingly, all input images are generally resized to a fixed resolution of 256 × 256 in the training phase for existing research on infrared image generation to guarantee training efficiency and stable model convergence. Nevertheless, an obvious mismatch exists between such a training scheme and the native operating resolution of practical infrared thermal imagers. Higher native resolutions are usually delivered by commercial infrared imagers compared with the training configuration. For instance, the shipborne FLIR-M364C thermal imager and the HW-TI100F3 series from Huawang Intelligence are equipped with uncooled vanadium oxide microbolometer infrared detectors of 640 × 512 resolution. The DLE1024 amorphous silicon uncooled focal plane array detector from Dali Technology supports an array output of up to 1024 × 768, whose aspect ratio also differs noticeably from that of the training data. If the model is overly sensitive to variations in input resolution, its direct deployment on real infrared sensors will be severely limited. It should be noted that the ConvNeXtV2 encoder has a fixed total downsampling factor of 32. For valid forward propagation, both the height and width of input images must be integer multiples of 32. Inputs failing to satisfy this dimension constraint cannot be fed into the network for infrared generation. Therefore, we select 640 × 512 and 1024 × 768 as test inputs, since both resolutions meet the tensor dimension requirement and correspond to typical native resolutions of commercial thermal imagers. We retain the model weights trained on 256 × 256 images without fine-tuning, to qualitatively verify the cross-resolution generalization performance of the proposed algorithm.

**Figure 12:**
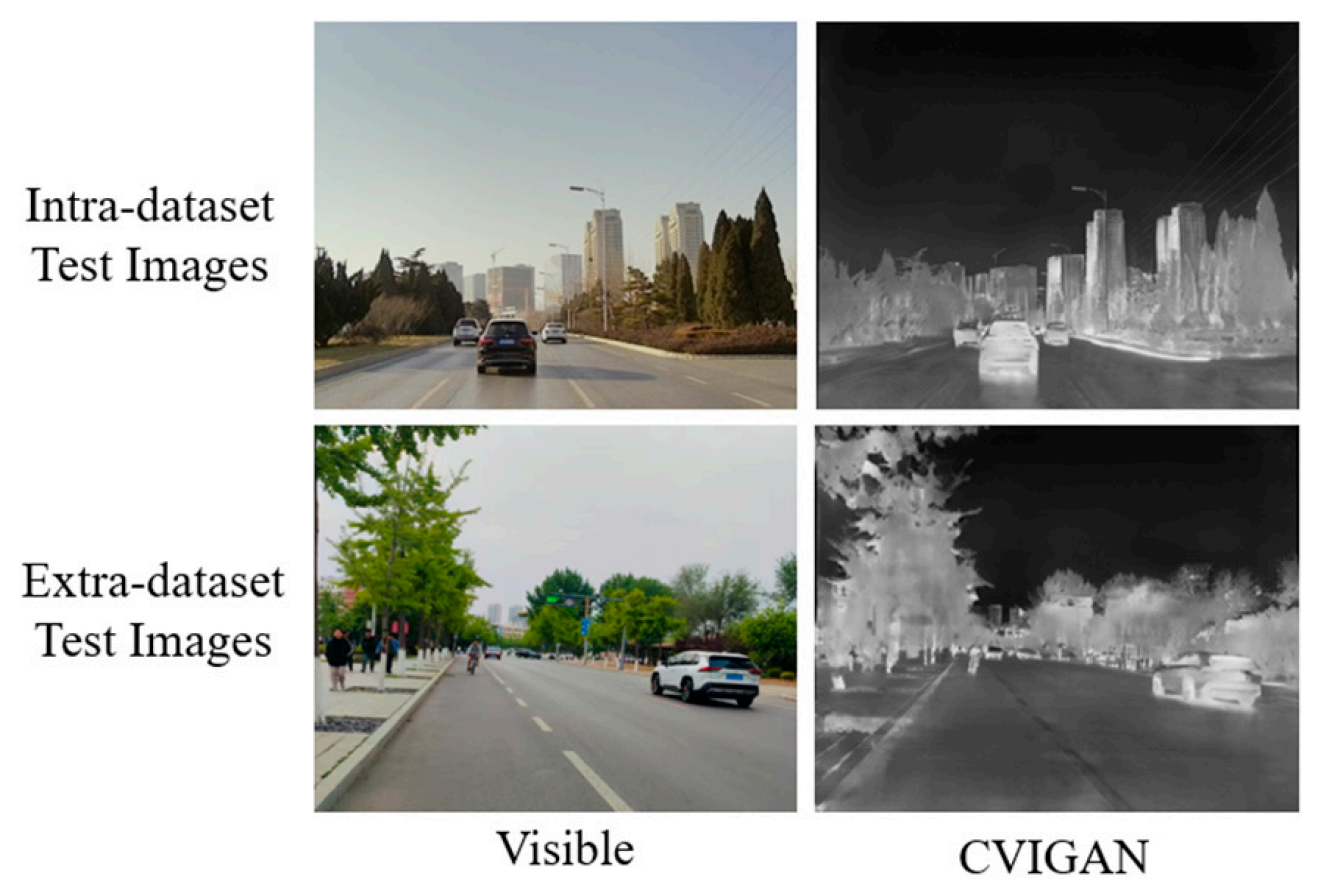
Test results at a resolution of 640 × 512.

**Figure 13:**
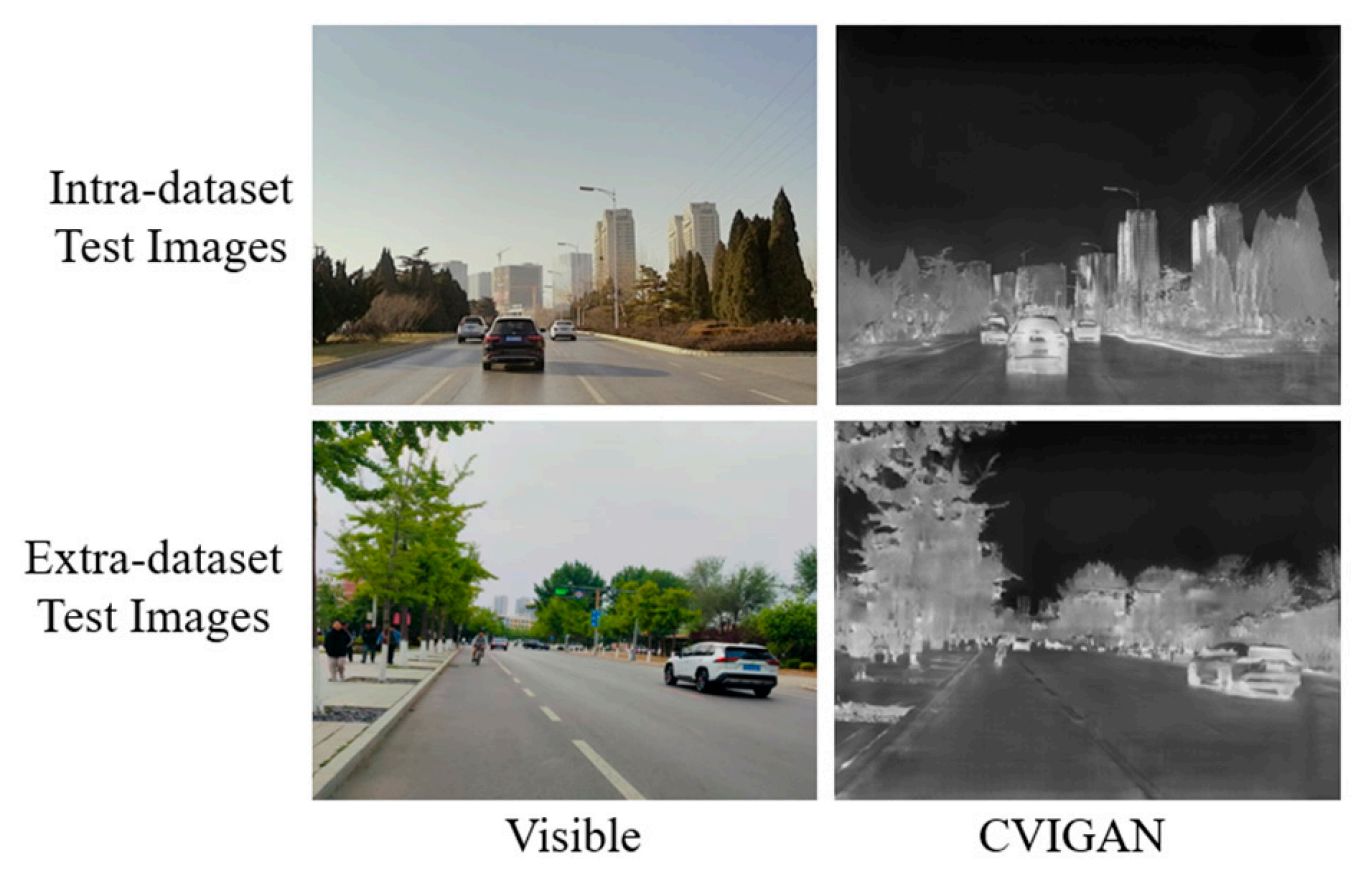
Test results at a resolution of 1024 × 768.

To comprehensively investigate the adaptability of the algorithm to varying input resolutions, the resolution generalization performance of the proposed CVIGAN is evaluated under two settings: intra-dataset testing and out-of-dataset testing. As illustrated in Figure 12 and Figure 13, the visualization comparisons with two different supported resolutions reveal that CVIGAN can reconstruct visually plausible infrared patterns for vehicles, high-rise buildings, roadside vegetation, and other objects for urban road scenes within the training distribution regardless of the input resolution. The contours of objects are well layered without large-area blurring, object deformation, or texture loss. For real captured road images outside the training dataset with more complex scene elements, the model still maintains reasonable generation performance. Complete and distinguishable infrared contours of pedestrians, cyclists, traffic signs, and moving vehicles on pavements are preserved. No detail collapse or excessive artifacts are observed for high-resolution inputs. Visual comparison of the two groups of images demonstrates that within the supported resolution range, the model can produce reasonable infrared outputs for these two high-resolution inputs without retraining. These qualitative results show that CVIGAN has certain cross-resolution generalization potential for these commonly used sensor resolutions. The method can adapt to visible-light acquisition devices with these specifications and partly ease deployment constraints when deploying the model onto practical infrared sensors. However, the model cannot perform inference for arbitrary image sizes that violate the multiple constraint of the encoder.

## 4. Conclusions

To overcome the limitations of existing unsupervised visible-to-infrared cross-domain translation networks, this paper proposes CVIGAN, a ConvNeXtV2-based generative adversarial network equipped with multi-scale frequency-aware attention for high-quality infrared image generation from unpaired visible data. To remedy blurred outlines and missing small objects caused by inadequate receptive fields and lost shallow semantics in traditional residual encoders, we construct a ConvNeXtV2 generator embedded with the multi-scale frequency-aware feature enhancement block (MFEB). Utilizing large convolution kernels and Global Response Normalization (GRN), the network suppresses color and illumination interference from visible images. A dual-branch frequency-aware attention fuses high frequency-aware and low frequency-aware multi-scale features to achieve reliable infrared visual reconstruction. A three-branch multi-scale discriminator extracts local texture and global scene information, where multi-resolution branches constrain edge features and grayscale distribution to realistic infrared visual patterns. A weighted composite loss combining least-squares adversarial loss, multi-layer PatchNCE contrastive loss, unidirectional cycle-consistency loss, and identity loss mitigates target misalignment, texture distortion, and brightness deviation.

Experiments on M3FD, AVIID3, self-built maritime SCHUAN dataset, and out-of-distribution campus datasets show that CVIGAN generates visually plausible infrared patterns for vehicles, buildings, and ships without grid artifacts, dislocation, or blurring, outperforming mainstream unsupervised translation methods quantitatively. Ablation studies validate the ConvNeXtV2 generator as the core performance booster, while contrastive loss and identity loss reduce distortion and preserve fine details. Cross-scene and cross-resolution tests demonstrate favorable robustness within supported input sizes: the model produces reliable infrared images on unseen maritime and campus scenes, and can process 640 × 512 and 1024 × 768 high-resolution industrial inputs without retraining, provided that both the height and width of input images are integer multiples of 32, which matches the fixed total downsampling factor of the ConvNeXtV2 encoder.

Downstream YOLOv5 detection experiments verify that synthesized infrared images enrich discriminative target features and improve detection accuracy, verifying the practical application potential. Future work will optimize reconstruction of distant and weak targets under heavy sea fog, strong backlight, and low-light conditions, and introduce meteorological priors to improve visual generation quality in extreme weather.

## Figures and Tables

**Table 1:** Algorithm comparison on the M3FD dataset.

Algorithm	SSIM	PSNR	LPIPS	FID
GLANET	0.5342	15.57	0.3803	141.10
DCLGAN	0.6145	18.02	0.2894	123.68
SimDCL	0.6102	17.35	0.3098	130.36
CUT	0.5833	17.97	0.2774	96.22
CycleGAN	0.5774	18.17	0.2460	105.77
UNSB	0.5138	19.67	0.2391	62.86
DR-AVIT	0.5024	13.98	0.5356	191.42
Ours	0.7139	23.37	0.1206	64.55

**Table 2:** Algorithm comparison on the AVIID3 dataset.

Algorithm	SSIM	PSNR	LPIPS	FID
GLANET	0.4583	17.49	0.2806	93.23
DC_LGAN_	0.5266	20.13	0.2202	68.86
SimDCL	0.5441	20.66	0.2005	61.82
CUT	0.4611	18.35	0.2716	76.25
CycleGAN	0.3807	15.92	0.3545	73.77
UNSB	0.5138	19.67	0.2391	62.86
DR-AVIT	0.3675	14.04	0.4696	100.42
Ours	0.5710	21.72	0.1700	58.48

**Table 3:** Algorithm comparison on the SCHUAN dataset.

Algorithm	SSIM	PSNR	LPIPS	FID
GLANET	0.6175	23.06	0.2214	97.20
DCLGAN	0.6336	24.81	0.2643	88.10
SimDCL	0.6317	23.27	0.2728	95.63
CUT	0.6234	23.42	0.1862	72.44
CycleGAN	0.3705	11.96	0.5001	86.31
Ours	0.6623	26.03	0.1539	54.84

**Table 4:** Ablation experiment data table on the M3FD dataset.

Generator	Discriminator	Nce Loss	Cycle Loss	Idt Loss	GAN(LS) Loss	SSIM	PSNR	LPIPS	FID
×	√	√	√	√	√	0.6080	19.70	0.2691	124.40
√	×	√	√	√	√	0.6712	20.78	0.1707	74.12
√	√	×	√	√	√	0.7091	23.21	0.1444	75.25
√	√	√	×	√	√	0.7109	23.32	0.1317	72.18
√	√	√	√	×	√	0.7091	23.22	0.1304	68.93
√	√	√	√	√	×	0.6864	22.44	0.1355	69.83
√	√	√	√	√	√	0.7139	23.37	0.1206	64.55

**Table 5:** Object detection results on synthetic infrared images from the Campus dataset.

Algorithm	Precision	Recall	Map50	Map50–95
Pix2Pix	0.545	0.625	0.522	0.274
Pix2Pix-MRFFF	0.570	0.497	0.431	0.180
EigenGAN	0.327	0.312	0.260	0.136
DCLGAN	0.572	0.375	0.402	0.202
SimDCL	0.782	0.250	0.271	0.142
GlANET	0.161	0.250	0.081	0.011
CycleGAN	0.810	0.449	0.449	0.200
CUT	0.414	0.375	0.308	0.133
Big-CycleGAN	0.764	0.375	0.468	0.197
DR-AVIT	0.349	0.188	0.167	0.057
Ours	0.845	0.688	0.717	0.374

## Data Availability

The original data presented in the study are openly available in the M3FD Dataset at https://github.com/dlut-dimt/TarDAL (accessed on 15 June 2025). Access to the AVIID-3 dataset is available upon reasonable request from the authors of the AVIID dataset.
